# The inter-annual variability of heat-related mortality in nine European cities (1990–2010)

**DOI:** 10.1186/s12940-018-0411-0

**Published:** 2018-08-08

**Authors:** Matteo Scortichini, Francesca de’Donato, Manuela De Sario, Michela Leone, Christofer Åström, Ferran Ballester, Xavier Basagaña, Janos Bobvos, Antonio Gasparrini, Klea Katsouyanni, Timo Lanki, Bettina Menne, Mathilde Pascal, Paola Michelozzi

**Affiliations:** 1 Environmental Epidemiology Unit, Lazio Regional Health Service ASL Roma 1, Rome, Italy; 2 Department of Public Health and Clinical Medicine, Umeå University, Umeå, Sweden; 3 FISABIO–Universitat Jaume I–Universitat de València Joint Research Unit of Epidemiology and Environmental Health, Valencia, Spain; 4 Spanish Consortium for Research on Epidemiology and Public Health CIBERESP, Madrid, Spain; 5 ISGlobal, Centre for Research in Environmental Epidemiology (CREAL), Barcelona, Spain; 6 Universitat Pompeu Fabra (UPF), Barcelona, Spain; 7 National Institute of Environmental Health, Budapest, Hungary; 8 Department of Social and Environmental Health Research, London School Hygiene and Tropical Medicine, London, UK; 9 Department of Hygiene, Epidemiology and Medical Statistics, Medical School University of Athens, Athens, Greece; 10 Department of Primary Care & Public Health Sciences and Environmental Research Group, King’s College London, London, UK; 11 Environmental Health Unit, National Institute for Health and Welfare, Kuopio, Finland; 12 Institute of Public Health and Clinical Nutrition, University of Eastern Finland, Kuopio, Finland; 13 WHO Regional Office for Europe, Copenhagen, Denmark; 14 Department of Environmental Health (DSE), Santé Publique France, Saint Maurice, France

**Keywords:** Heat, Mortality, Temporal trends, Bayesian models, Time factors, Attributable risk

## Abstract

**Background:**

The association between heat and daily mortality and its temporal variation are well known. However, few studies have analyzed the inter-annual variations in both the risk estimates and impacts of heat. The aim is to estimate inter-annual variations in the effect of heat for a fixed temperature range, on mortality in 9 European cities included in the PHASE (*Public Health Adaptation Strategies to Extreme weather events)* project for the period 1990–2010. The second aim is to evaluate overall summer effects and heat–attributable deaths for each year included in the study period, considering the entire air temperature range (both mild and extreme temperatures).

**Methods:**

A city-specific daily time-series analysis was performed, using a generalized additive Poisson regression model, restricted to the warm season (April–September). To study the temporal variation for a fixed air temperature range, a Bayesian Change Point analysis was applied to the relative risks of mortality for a 2 °C increase over the 90th percentile of the city-specific distribution. The number of heat attributable deaths in each summer were also calculated for mild (reference to 95th percentile) and extreme heat (95th percentile to maximum value).

**Results:**

A decline in the effects of heat over time was observed in Athens and Rome when considering a fixed interval, while an increase in effects was observed in Helsinki. The greatest impact of heat in terms of attributable deaths was observed in the Mediterranean cities (Athens, Barcelona and Rome) for extreme air temperatures. In the other cities the impact was mostly related to extreme years with 2003 as a record breaking year in Paris (+ 1900 deaths) and London (+ 1200 deaths).

**Conclusions:**

Monitoring the impact of heat over time is important to identify changes in population vulnerability and evaluate adaptation measures.

**Electronic supplementary material:**

The online version of this article (10.1186/s12940-018-0411-0) contains supplementary material, which is available to authorized users.

## Background

In the last 10 years, the scientific evidence regarding the health impact of high air temperatures and heat waves has become well established both throughout Europe and in the international context [1–5]. Researchers have considered different air temperature exposures [6], refined statistical techniques to better characterize the temperature-mortality relationship [7] and accounted for the potential effect modifiers that might explain the heterogeneous effects among local populations or between cities/countries [8]. Multicity and multinational studies have been carried out with a common methodological approach consolidating the evidence in terms of heat and cold effects on health and in particular on the geographical differences in the risk estimates and in terms of the impacts (attributable fraction/deaths) [4].

Time trends in the temperature-mortality association have also been studied, addressing the potential factors which might contribute to the variation in effects such as changes in local climate, population susceptibility and the introduction of adaptation measures [9]. Studies comparing discrete time periods (5, 10 years) [9–12] or evaluating the overall trend [13–15] have provided evidence of such changes. Temporal variations have also been studied in relation to extreme events against a reference period or single years [16, 17] and in before-after studies comparing time periods before and after an event which might have caused a modification in the relationship [3, 18, 19]. A reduction in heat-related mortality estimates over time was observed in areas with different climatic and population characteristics and this can be attributed to the introduction of population adaptation plans, individual protection measures and more general technological improvements (e.g. air conditioning, cooling) [3, 9]. A recent European study conducted on the same cities showed a reduction in heat-related risks in mediterranean cities after 2003 [3].

Limited studies looking at the temporal variations in terms of both the overall trend and inter-annual variations have been carried out [15, 20]. The main determinants of heat-related mortality burden in a given year are the meteorological conditions a local population is exposed to. Extreme events which have had a significant burden in terms of mortality in the recent decades in Europe, include the summer of 2003 [2] and more recently 2015 [21]. Because of the increase in frequency and intensity of heat waves already observed in the last decades, and predicted for the future climate change scenarios [22], year-by-year variations in heat-related mortality, in addition to medium- and long-term changes need to be considered. In Europe, after summer 2003, the introduction of heat prevention plans may have improved awareness and adaptation among local populations. An increase in public awareness may also have occurred simply through greater media attention and the more frequent occurrence of severe heat waves in recent years [23]. Another important factor that influences heat-related mortality is previous winter season mortality; by affecting the same vulnerable population the burden in the following summer season is dependent on winter cold spells and the influenza season [24, 25]. Furthermore, considering the ageing of the European population and thus the potential increase in the number of vulnerable subjects in future years, it is important to evaluate the temporal change in both the effects and impacts of heat at the inter-annual scale. To date there is limited evidence on these aspects.

Within the EU project PHASE-*Public Health Adaptation Strategies to Extreme weather events* (www.phaseclimatehealth.eu), a first study was carried out to assess the change in heat vulnerability between two periods: before and after 2003 in European cities [3]. The current analysis is aimed at evaluating the temporal variation in heat-related mortality in nine European cities over a 20-year study period in the warm season (April–September). Specifically, the study will evaluate the temporal and inter-annual change in the risk response to a specific fixed temperature range by estimating the variations in the effect for a fixed air temperature interval. Secondly the study will provide summer season relative risks (RR) and impacts (in terms of heat–attributable deaths), considering both mild and extreme temperatures occuring in each summer. These estimates will describe how heat impacts vary from year to year.

## Methods

### Dataset

Data was collected for nine European cities (Athens, Barcelona, Budapest, Helsinki metropolitan area, Paris, London, Rome, Stockholm, Valencia), included in the PHASE project which are characterized by different climatic and socio demographic conditions. Each city provided daily mortality and meteorological data for the period 1990–2010 depending on availability. Details on city-specific datasets are provided in the previous paper by the same authors [3].

Briefly, the outcome data considered was daily mortality counts for all natural causes (ICD9: 1–799) while exposure data was daily mean air temperature (°C) calculated as the average of 3-hourly air temperature readings in the 24 h period from airport or city monitoring stations.

### Statistical methods

In the PHASE project a preliminary analysis was conducted to select the best exposure, lag structure and confounders for modelling the relation between air temperatures and mortality and is described elsewhere [3].

To estimate the effect of heat on mortality a city-specific generalized additive regression model with a Poisson distribution, allowing for overdispersion during the warm season (April – September), was carried out:

$$ \mathit{\log}\left[E\left({Y}_i\right)\right]=\alpha + tensor\left({Tmean}_n, Time\right)+s(dos)+ dow+ hol+ wdd $$

Where: *Y*_*i*_ is the number of deaths in the day *i*; *Tmean* is a moving average of the current day and previous *n* days of the daily mean air temperature (Tmean) where *n* is defined as the city-specific maximum significant lag; *Time* is the progressive count of days in the study period; *dos* is the day of season (values from 1 to 183) fitted with a spline with 6 dof (one for each month) to control for seasonal trends; *dow* and *hol* are categorical variables for day of the week and holidays respectively and *wdd* is the average daily mortality observed in the previous winter (October to March) fitted as a linear term to account for previous year winter mortality [24, 25]. This last variable was added in the model after running a sensitivity analysis on the effect modification of previous winter mortality on the strength of summer temperatures’ effect (data not shown).

To analyze how the effect varies over time, a tensor product of the “interaction” between time and exposure was defined in order to obtain time-varying estimates. We defined two marginal basis functions [26]:

$$ f\left({Tmean}_n\right)=\sum \limits_{i=1}^{n_1}{b}_i\left({Tmean}_n\right){\beta}_i $$

$$ g(Time)=\sum \limits_{l=1}^{n_2}{a}_l(Time){\alpha}_l $$

where *b*_*i*_ is the i^th^ basis for temperature, *a*_*l*_ the l^th^ basis for time, *β*_*i*_ and *α*_*l*_ are unknown parameters. If we allow *f*(*Tmean*_*n*_) (i.e. its parameters *β*_*i*_) to vary smoothly with *Time*:

$$ {\beta}_i(Time)=\sum \limits_{l=1}^{n_2}{a}_l(Time){\alpha}_{il} $$

we obtain a bivariate tensor interaction smooth:

$$ Tensor\left({Tmean}_n, Time\right)=\sum \limits_{i=1}^{n_1}\sum \limits_{l=1}^{n_2}{a}_{il}(Time){\alpha}_{il}{b}_i\left({Tmean}_n\right) $$

A cubic regression spline with 2 equally spaced knots was used as a smooth function for air temperature, while for time we defined a cubic regression spline with a 1 dof for each year in the study period, to allow the temperature-mortality association to vary per annum.

To address the first aim and estimate the temporal variation in mortality for a fixed interval, the percent (%) change in mortality for an increase of 2 °C above the city-specific 90th percentile of the mean air temperature distribution (study period comprised between 1990 and 2010) was calculated in each city for each summer. In order to detect significant changes in the risks of mortality due to heat over time, we applied a Bayesian Change Point (BCP) model [27] to the daily series of risk estimates. Under the hypothesis that a given time series is a sequence of contiguous blocks, such that the mean value is constant within each block, for every observation the algorithm estimates the posterior probability of being a change point, i.e. the boundary between two blocks. We ran a Markov chain Monte Carlo change point algorithm (MCMC) to identify the suitable change points by estimating the probability of a change as a function of time. For each iteration a value of k, the suitable change point, was selected and two gaussian distributions with different means were estimated on the two series of estimates obtained, on the original logarithmic scale:

$$ {\beta}_i\sim Gaussian\left({\mu}_1,\sigma \right)\ i=1,\dots, k $$

$$ {\beta}_i\sim Gaussian\left({\mu}_2,\sigma \right)\ i=k+1,\dots, n $$

The Markov Chain guarantees that the k value selected at each time step depends only on the values of k, *μ*_1_ and *μ*_2_ estimated on the previous step. At the end of the iterating process, the posterior distributions *f*(*μ*_1_, *β*) and *f*(*μ*_2_, *β*) can be estimated, as well as the probability of each k being a change point, *p*(*k*| *μ*_1_, *μ*_2_, *β*). Since the algorithm needs some iteration to start to converge towards the final result, the first iterations are not considered in the estimate of the posterior distributions (burn-in).

Each MCMC simulation consisted of 11,000 iterations (first 1000 as burn-in). To detect “significant” change-points we used an approach similar to the one adopted by Khaliq and co-authors [28]: in absence of change-points, the time-varying coefficients arising from the model should behave like a unique Gaussian distribution. 1000 random time series following a Gaussian distribution were generated, with parameters equal to the Mean and Variance of the distribution of estimated coefficients and on every series we ran the BCP algorithm. Confidence intervals were built by selecting the 975th largest posterior probability for every coefficient and then applying a smoothing function to the results. We used the BCP algorithm to detect changes in the effect estimates trend over time, but also to identify extraordinary heat events in each city.

Secondly, to account for changes in the overall impact of heat during each summer, we estimated the % change in mortality from the city-specific threshold (Tref) to the maximum observed air temperature value. Tref corresponds to the air temperature value above which mortality started to increase (turning point); this was considered constant over time after visual inspection of the annual curves and model results. We also calculated the number of attributable deaths for each summer as carried out by Baccini et al. [29]. Given a coefficient β_ijT_ expressing the effect of temperature on mortality estimated from the tensor with respect to the Tref value, where *i* represents the day of the year, *j* the year and T the temperature over Tref, the annual daily deaths attributable to heat were calculated as:

$$ {AD}_j=\sum \limits_i{y}_{ij}\ast \left(1-{e}^{-{\beta}_{ij T}}\right) $$

Where *β*_*ijT*_=0 if T ≤ Tref.

Furthermore, to evaluate the impact of extreme and mild heat separately, we calculated the number of attributable deaths for mild heat (from Tref to the 95th percentile of the overall mean air temperature city specific summer distribution) and for extreme heat (from the overall 95th percentile to the maximum value observed in each summer).

## Results

A first description of the data highlights that mean air temperatures show a slight increasing trend in most cities, while in terms of mortality a reduction in daily deaths is observed in several cities (ranging from − 0.1 mean annual daily deaths in Barcelona to − 2.7 in London) (Table 1). In Athens and Rome an increase was observed (+ 0.6 and + 0.2 mean annual daily deaths respectively), no change was shown in Helsinki and Valencia. Mean daily deaths per year over the 20 year study period are shown in Additional file 1: Table S1. These results are confirmed when looking at the city-specific annual series of boxplots for both air temperature and daily deaths (see Additional file 2: Figures S2-S10).

**Table 1:** Description of study period, total mortality and mean temperature in nine European cities

City	Study Period	Total deaths		Mean Temperature (C°)	
		mean daily value	mean trend	mean daily value	mean trend (°C)
HELSINKI^a^	1990–2010	17	0.0	12.5	+ 0.09
STOCKHOLM	1990–2010	29	−0.1	13.2	+ 0.07
LONDON	1990–2010	144	−2.7	15.4	+ 0.03
PARIS	1991–2009	108	−1.4	17.1	+ 0.05
BUDAPEST	1992–2010	65	−1.0	18.4	+ 0.03
ROME	1992–2010	53	+ 0.2	20.9	+ 0.06
BARCELONA	1991–2009	36	−0.1	20.8	+ 0.14
VALENCIA	1994–2010	15	0.0	22.3	−0.02
ATHENS	1992–2010	73	+ 0.6	24.1	+ 0.07

^a^Helsinki metropolitan area includes 4 cities: Helsinki, Espoo, Vantaa and Kauniainen

**Fig. 1:**
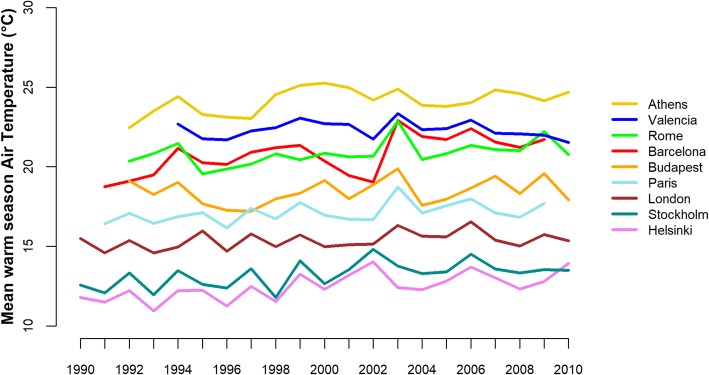
Warm season mean temperatures by year, in the nine European cities over the period 1990–2010

Figure 1 shows the geographical heterogeneity in average summer air temperatures, with higher values among the Mediterranean cities (20–24 °C) and lower values in Scandinavian cities (12–13 °C). Furthermore, air temperatures vary throughout the 20 year period with a slight increasing trend in all cities. Peak hot summers can be easily detected in each city as well as extreme events at the European scale, such as 2003. City-specific summer air temperatures (percentile at Tref, number of days above Tref, reference air temperature at 95th percentile and corresponding percentiles in each year, maximum air temperature) are provided in Additional file 1: Table S1.

**Fig. 2:**
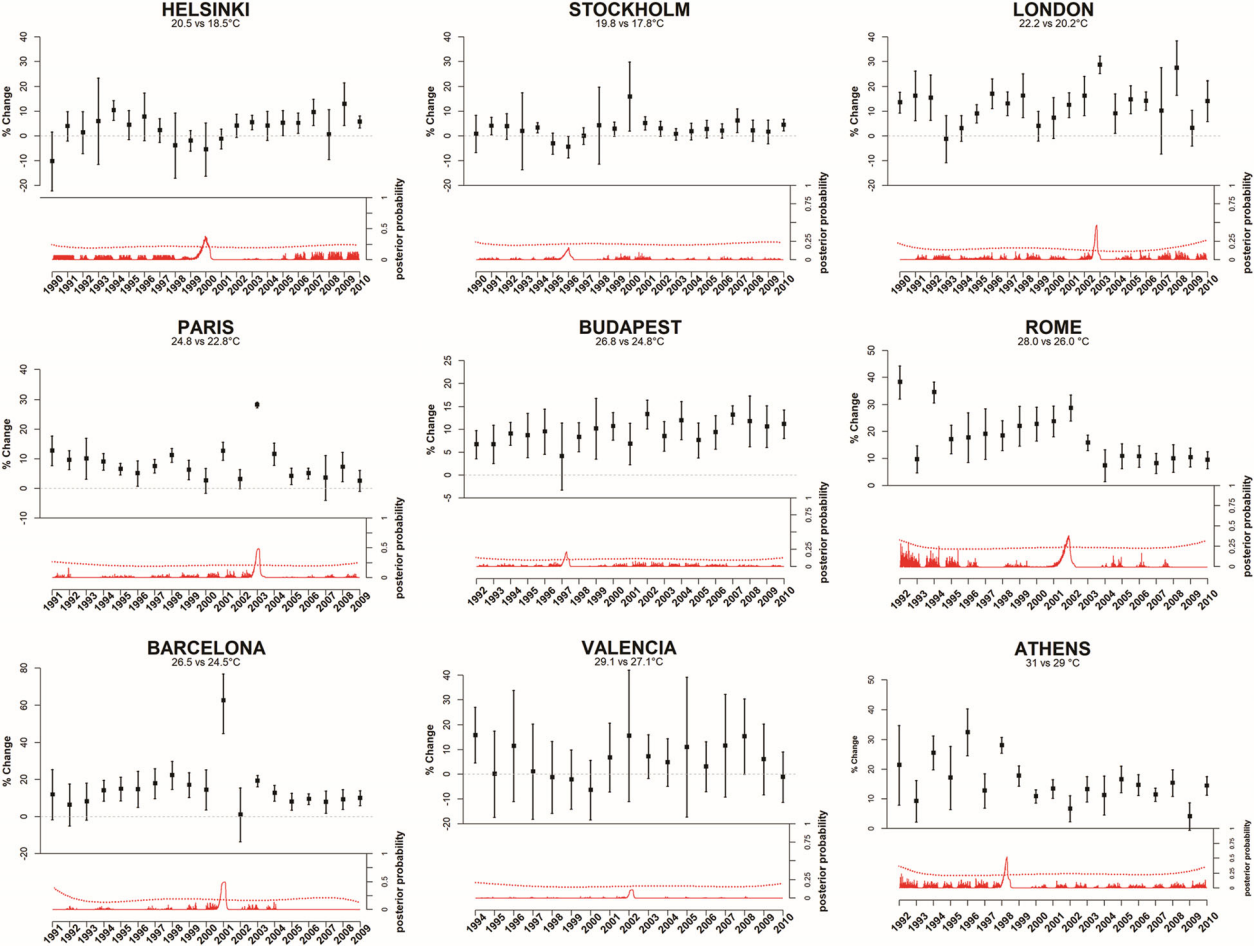
Heat-related mortality effects in nine European cities for each summer (period 1990–2010). top panel: Effects expressed as percent change in mortality for a 2 °C increase in mean temperature over the threshold; bottom panel: posterior probability of being a change point of each estimated observation from Bayesian Change Point analysis. The red dotted lines represent the statistical significance

Figure 2 shows the percent change in daily mortality for a fixed air temperature range (2 °C increase in mean air temperature above the 90th percentile) in each summer (upper figure) and the probability of a change in the mortality estimates (lower figure). A geographic variability in the estimates of heat-related mortality for a fixed air temperature range is shown, with a greater effect of heat in Mediterranean cities and lowest in Scandinavian cities. In all cities, year-to-year variations can also be detected, either for single peak summers with very high estimates, annual fluctuations around similar values, or with more distinct patterns of change throughout the time series studied. Summers with few hot days contributing to the heat risk have much wider intervals.

A variation in the temporal trend can be observed in Fig. 2 and was identified by a peak in the BCP probability graph for Athens, Rome and Helsinki; with a decrease in the effect in Rome (after 2004) and in Athens (from 2000) and a rise in Helsinki (from 2002). In Barcelona, London and Paris single peak years with extremely high effect estimates (62, 29, 28%, respectively) were detected by the BCP algorithm, specifically 2003 in London and Paris and 2001 in Barcelona. In Budapest, significant effects were estimated for most summers, with a fluctuation around similar values. In Stockholm the effects, although not significant for most summers, became more consistent in more recent years. Effect estimates in Valencia were not significant for most summers and no temporal change or peak year was identified.

**Fig. 3:**
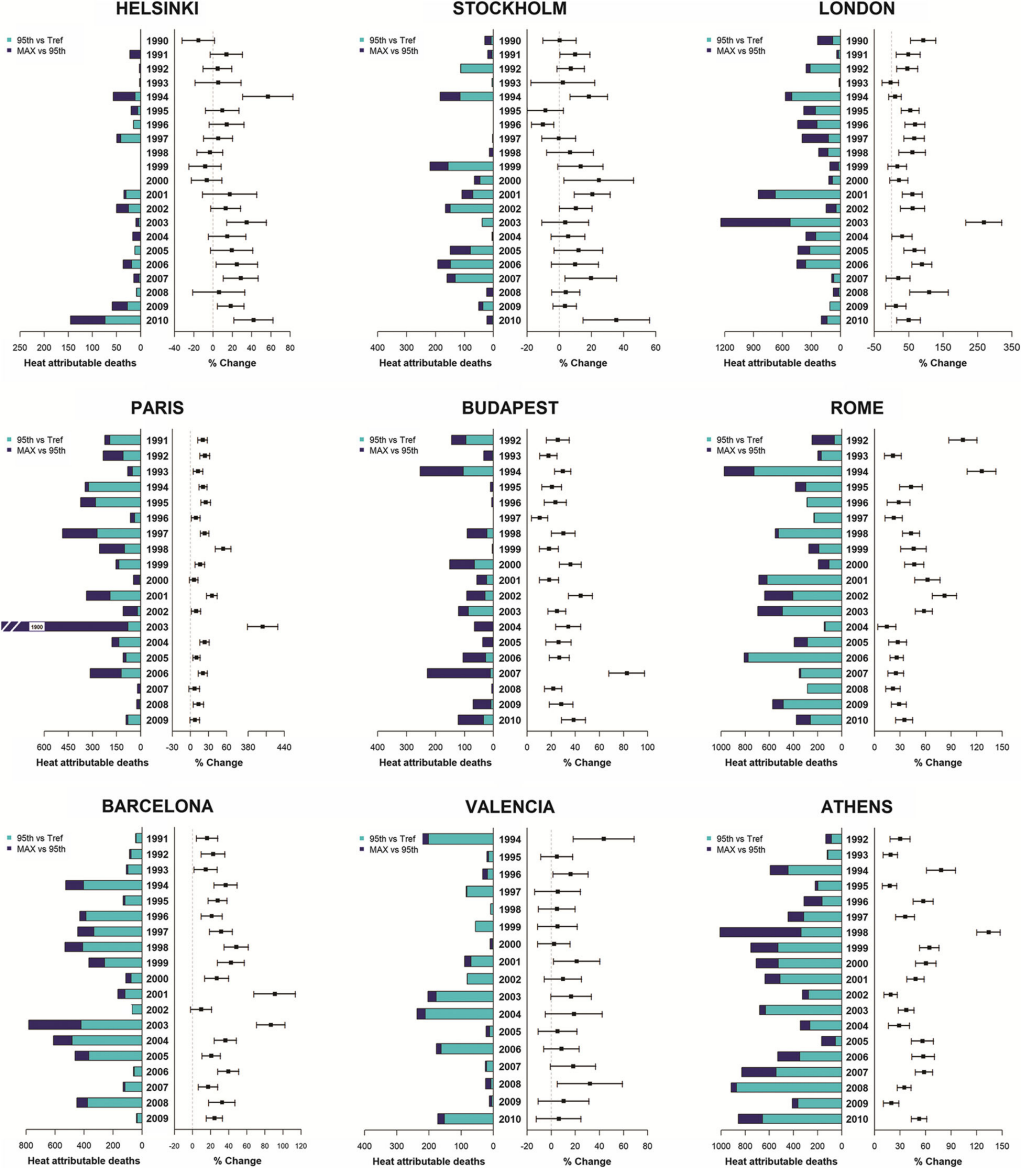
Overall heat-related mortality effects and impacts in nine European cities for each summer (period 1990–2010). Right hand-side: risk estimates attributable to heat (from Tref to maximum); left hand-side: heat-attributable deaths to mild heat (from Tref to 95th percentile, light blue) and to extreme heat (from 95th percentile to maximum, dark blue)

Figure 3 illustrates the overall summer relative risk estimates (right hand side) and attributable deaths (left hand side), for mild (light blue) and extreme heat (dark blue), for each summer. When describing the annual effects of heat, considering both risk estimates and impacts, it is clear that there was a great inter-annual variability, in particular for attributable deaths. The greatest impact of heat in terms of attributable deaths was observed among the Mediterranean cities (Athens, Barcelona and Rome) for both mild and extreme temperatures, while in the other cities the impact was mostly related to extreme heat. In Athens, when considering summer risk estimates, the declining trend was less clear compared to fixed temperature estimates. In Rome, the declining trend after 2004 was confirmed also when considering summer effect estimates (Fig. 3); however when considering attributable deaths the greatest reduction was for extremes temperatures. The overall summer analysis for Barcelona showed more variability compared to the fixed interval analysis.

Considering extreme events, the year with the greatest effect estimates and impacts in terms of extreme heat was 1998 in the Mediterranean cities and in Paris and London. Two summers were exceptional in terms of effects, but only 2003 had an important impact on mortality especially for extreme air temperatures (362 heat-related deaths). In London and Paris, the overall summer analysis confirmed findings from the previous fixed temperature estimates; with 2003 standing out as the record breaking year both in terms of risks (percent change: London + 280% and Paris + 400%) and attributable deaths for extreme temperatures (London: + 1200 and Paris: + 1900 death counts). In Budapest, summer air temperature effects were significant in all summers with a considerable number of attributable deaths for extreme heat rather than for mild temperatures as observed in other cities. Conversely, to what was observed in the fixed interval analysis, summer 2007 stands out as a peak year in Budapest with very a high heat effect estimate (+ 80%) and impacts (217 attributable deaths on days with extreme temperatures). In Helsinki, although the effects and impacts of heat in summer were mostly non-significant, the rising trend was confirmed and the last two summers in study had the highest impacts in terms of extreme temperature attributable deaths. In Stockholm, the overall summer analysis showed a greater inter-annual variability even if non-significant effect estimates were estimated. In both Stockholm and Helsinki, 2010 was an exceptional year in terms of heat-related impacts. The overall summer analysis confirmed non significant effects for most summers in Valencia. Conversely to other Mediterranean cities, the impact of extreme temperatures in Valencia was limited compared to that observed for mild temperatures.

## Discussion

The use of a flexible method to simultaneously estimate the time trends in heat-related mortality and the inter-annual changes due to variations in exposure or population vulnerability can help better understand the dynamics of heat-related deaths and the factors influencing it. With respect to previous studies on the same dataset comparing the effect of heat in two different periods [3], or assuming a linear trend of the effect of temperatures [14], this methodology allows a more comprehensive evaluation of the inter-annual variability of the effect of high temperatures.

Inter-annual variability is infleunced by exposure and the size and characteristics (demographic phenomena and clinical conditions) of the pool of subjects most at risk. Furthermore, considering annual fluctuations in heat-related deaths, it is important to consider seasonal stressors that impact on mortality, especially among the pool of susceptible subgroups. Typically, previous winter mortality has been shown to influence the impact of heat in the following summer [24, 25, 30, 31]. High mortality winters may indeed deplete the number of high-risk individuals thus reducing the impact of heat waves the following summer, as observed in winter 2014/2015 when influenza-related deaths among the elderly peaked in many European countries [21, 32]. A sensitivity analysis was conducted, stratifying by high and low previous winter mortality, and the effect of heat was stronger in summers with a low previous winter mortality (data not shown) especially for Athens, Rome, Budapest and Helsinki. A geographical pattern in terms of years with high winter deaths was observed in the European cities included in our study, suggesting that larger scale phenomena, such as seasonal flu epidemics, can also influence mortality. The same susceptible subgroups are those mostly affected during influenza outbreaks. In recent years, a decline in flu vaccination coverage has been reported [33], potentially inflating the pool of high risk individuals. Furthermore, seasonal impacts of flu viruses also depend on vaccine composition, if there is a mismatch with respect to the dominant virus subtype, the actual coverage will be lower [32].

Speculative explanations of the observed inter-annual variability can be attributable to social or economic drivers, exposure and concomitant environmental exposures such as air pollution (traffic or forest fire emission sources). The study identified summers with extreme exposure such as 2003 that had an exceptional impact on mortality. In Paris, the death toll was outstanding, with 1900 excess deaths on the entire summer (almost the totality were attributable to extreme heat), even higher than observed in a previous French study [34] where + 149% excess deaths were observed only from 1st-20th August 2003. Similarly, in London summer 2003 was denoted as exceptional both in terms of exposure and in heat-related impacts, confirming previous findings [35, 36]. In Barcelona, summer of 2001 was not extremely hot but recorded very high effect estimates: probably influenced by wildfires in the same days as the heat wave [37]. Similarly, the exceptional impact recorded in 1998 in Athens may also be affected by the forest fires episodes recorded during that summer [38]. Exceptional heat wave events explained at least in part the oversized percent change and attributable deaths due to extreme heat in the whole summer in Budapest in 2007 [39]. In Budapest, a high number of in-hospital deaths was observed during the heat wave of 2007, possibly due to the lack of air conditioning in hospital wards [40]. Another exceptional event in northern Europe was summer 2010 [41]. These events had a significant impact in Helsinki and Stockholm. In Helsinki, the greater impact may also be explained by the synergistic effect of heat waves and air pollution. In fact, long-range transport of pollutants from forest fires have been shown to have an effect on health outcomes [42].

The vulnerability of local populations to heat may increase as a consequence of climate change predicted for Europe [22] especially in those countries where these events are rare. An increasing trend in heat-related mortality in both the effect and impacts of heat was detected in Helsinki, in parallel with the rise in average summer air temperatures also described by Irannezhad [43]. Another factor, which may enhance the vulnerability to heat in future years, especially in terms of attributable deaths, is the ageing of the European population [44] and the subsequent rise in the prevalence of chronic disease which will amplify the pool of susceptible individuals most at risk during extreme heat [45].

Studies looking at time trends of the temperature-mortality association, have suggested that the introduction of public health adaptation measures, the improvement of health care systems, the increase in air conditioning usage and the improved population awareness have helped reduce the burden of health [13, 20, 46–51]. Public health heat prevention plans have been widely introduced in the US since the 1990’s and more recently after 2003 in European countries [52, 53]. The WHO defined guidance documents with a set of core elements necessary for the prevention of heat-related health effects [54]. In Rome, the introduction of a heat prevention plan from 2004 onwards which includes GP active surveillance on susceptible elderly subgroups during heat waves may have contributed to the reduction in the impact of heat for extreme temperatures [3, 18, 55]. Although the temporal trend is less clear for London, a reduction in extreme heat attributable deaths was observed. This could be related to the introduction of the UK heat plan in 2004 as suggested by Green et al. [35] as well as a progressive decline in cardiovascular deaths in the last 30 years [56]. Previous studies conducted in England and Wales on a previous time period (1976–2005) estimated a long-term increase in heat-related mortality of 0.7 deaths per million per year [57]. In Budapest, although the heat health warning system was introduced in 2005, a decline in heat-related excess mortality has not been observed [40].

The study evaluates the temporal variations in heat-related mortality. However, some strengths and weaknesses should be acknowledged. The study has the advantage of including nine major European cities and a 20-year study period comprising the 2003 extreme event and years in which adaptation measures were introduced across Europe. The study suggests the importance of considering both the temporal changes in risk estimates of heat as well as in the burden on local populations during the entire summer season as they can provide relevant complementary information. Although summer estimates of heat-related deaths for a single year are less robust due to the limited number of days in the study period, they can be a useful tool to make comparisons within a city over time. Furthermore, for modeling purposes, we were only able to select total mortality for all ages, as the tensor smoother requires a considerable number of daily outcome counts in order to converge. Another potential limitation worth mentioning is that estimates were not adjusted for air pollutants, due to data and model limitations. The primary interest here was to consider inter-annual variations within cities over time. The role of air pollutants as potential confounders and/or effect modifiers could be addressed in a further study on heat wave episodes.

The study allows to collect information in terms of inter-annual variations in the mortality burden related to heat which depend on the relative risk, but also on the specific air temperature range experienced by the local population, the occurrence of extreme events, the size of the pool of susceptible subgroups. The observed changes need to be taken into account when planning adpatation strategies at local or European scale, results suggest the need for a periodical update of heat-response plans and a progressive extension to regions not covered yet. Future research on heat-related vulnerability factors should address their role in explaining long-term temporal trends rather than inter-annual variations.

## Conclusions

In conclusion, the study shows that the association between heat and mortality has an inter-annual variability which depends on several concomitant factors, with summer temperatures and population vulnerability playing a key role. Therefore, future studies focusing on the burden of temperatures within a specific summer require data from both relative risk, which measures population response to heat, and the attributable risk, or burden. Annual fluctuations are important for both research and public health, especially when considering the increase in the frequency and intensity of extreme events predicted under future climate change. Adequate adaptation measures in the short-term and more structured mitigation policies in the long term are needed not only in the warmer Mediterranean countries but also in the cooler northern European regions, in order to potentially reduce present and future risks and improve responses in urban areas.

## Additional files

Additional file 1: **Table S1.** Temperature distribution and mean daily mortality by year, in the nine European cities (period 1990–2010). Reference temperature value (°C) and percentile, 95th percentile of overall summer temperature distribution (°C, used as the threshold for definition of mild and extreme heat), number of mild and extreme heat days, maximum temperature (°C) and mean daily mortality (number of deaths) for each city and for each year. (XLSX 17 kb)

Additional file 2: **Figures S2-S10.** Temperature and mortality distribution by year in the nine European cities (period 1990–2010). Boxplots of temperature and mortality for each city and for each year. (ZIP 934 kb)

## Abbreviations

BCP: Bayesian Change Point
EU: European Union
GP: General Practitioners
ICD9: International Classification of Diseases 9th Revision
MCMC: Markov chain Monte Carlo
PHASE: Public Health Adaptation Strategies to Extreme weather events
UK: United Kingdom
US: United Stated
WHO: World Health Organization

## References

[1] Baccini M, Biggeri A, Accetta G, Kosatsky T, Katsouyanni K, Analitis A, et al. Heat effects on mortality in 15 European cities. Epidemiology. 2008; 10.1097/EDE.0b013e318176bfcd 18520615

[2] D’Ippoliti D, Michelozzi P, Marino C, de’Donato F, Menne B, Katsouyanni K (2010). The impact of heat waves on mortality in 9 European cities: results from the EuroHEAT project. Environ Health.

[3] de’Donato FK, Leone M, Scortichini M, De Sario M, Katsouyanni K, Lanki T (2015). Changes in the Effect of Heat on Mortality in the Last 20 Years in Nine European Cities. Results from the PHASE Project. Int J Environ Res Public Health.

[4] Gasparrini A, Guo Y, Hashizume M, Lavigne E, Zanobetti A, Schwartz J (2015). Mortality risk attributable to high and low ambient temperature: a multicountry observational study. Lancet.

[5] Guo Y, Gasparrini A, Armstrong B, Li S, Tawatsupa B, Tobias A (2014). Global variation in the effects of ambient temperature on mortality: A systematic evaluation. Epidemiology.

[6] Barnett AG, Tong S, Clements ACA (2010). What measure of temperature is the best predictor of mortality?. Environ Res.

[7] Gasparrini A, Armstrong B, Kenward MG (2010). Distributed lag non-linear models. Stat Med.

[8] Jackson D, White IR, Thompson SG (2009). Extending DerSimonian and Laird’s methodology to perform multivariate random effects meta-analyses. Stat Med.

[9] Arbuthnott K, Hajat S, Heaviside C, Vardoulakis S. Changes in population susceptibility to heat and cold over time : assessing adaptation to climate change. Environ Health 2016;15 Suppl 1:33. 10.1186/s12940-016-0102-7 PMC4895245 26961541

[10] Petkova EP, Gasparrini A, Kinney PL (2014). Heat and mortality in New York City since the beginning of the 20th century. Epidemiology.

[11] Åström DO, Forsberg B, Edvinsson S, Rocklöv J (2013). Acute fatal effects of short-lasting extreme temperatures in Stockholm, Sweden: evidence across a century of change. Epidemiology.

[12] Carson C, Hajat S, Armstrong B, Wilkinson P (2006). Declining vulnerability to temperature-related mortality in London over the 20th century. Am J Epidemiol.

[13] Bobb JF, Peng RD, Bell ML, Dominici F (2014). Heat-related mortality and adaptation to heat in the United States. Environ Health Perspect.

[14] Gasparrini A, Guo Y, Hashizume M, Kinney PL, Petkova EP, Lavigne E (2015). Temporal variation in heat-mortality associations: a multicountry study. Environ Health Perspect.

[15] Guo Y, Barnett AG, Tong S (2012). High temperatures-related elderly mortality varied greatly from year to year: important information for heat-warning systems. Sci Rep.

[16] Fouillet A, Rey G, Wagner V, Laaidi K, Empereur-Bissonnet P, Le Tertre A (2008). Has the impact of heat waves on mortality changed in France since the European heat wave of summer 2003? A study of the 2006 heat wave. Int J Epidemiol.

[17] Tan J, Zheng Y, Tang X, Guo C, Li L, Song G (2010). The urban heat island and its impact on heat waves and human health in shanghai. Int J Biometeorol.

[18] Schifano P, Leone M, De Sario M, de’Donato F, Bargagli AM, D’Ippoliti D (2012). Changes in the effects of heat on mortality among the elderly from 1998-2010: results from a multicenter time series study in Italy. Environ Health.

[19] Morabito M, Profili F, Crisci A, Francesconi P, Gensini GF, Orlandini S (2012). Heat-related mortality in the Florentine area (Italy) before and after the exceptional 2003 heat wave in Europe: an improved public health response?. Int J Biometeorol.

[20] Sheridan SC, Kalkstein AJ, Kalkstein LS (2009). Trends in heat-related mortality in the United States, 1975–2004. Nat Hazards Springer Netherlands.

[21] Michelozzi P, de’Donato F, Scortichini M, De Sario M, Asta F, Agabiti N (2016). On the increase in mortality in Italy in 2015: analysis of seasonal mortality in the 32 municipalities included in the Surveillance system of daily mortality. Epidemiol Prev.

[22] Qin D, Plattner G-K, Tignor M, Allen SK, Boschung J, Nauels A, IPCC (2013). Summary for Policymakers. Clim Chang 2013 Phys Sci Basis Contrib Work Gr I to Fifth Assess Rep Intergov Panel Clim Chang.

[23] Akompab DA, Bi P, Williams S, Grant J, Walker IA, Augoustinos M (2012). Awareness of and attitudes towards heat waves within the context of climate change among a cohort of residents in Adelaide, Australia. Int J Environ Res Public Health.

[24] Stafoggia M, Forastiere F, Michelozzi P, Perucci CA (2009). Summer temperature-related mortality: effect modification by previous winter mortality. Epidemiology.

[25] Qiao Z, Guo Y, Yu W, Tong S (2015). Assessment of short- and long-term mortality displacement in heat-related deaths in Brisbane, Australia, 1996-2004. Environ Health Perspect.

[26] Wood SN. Generalized Additive Models: an introduction with R COPYRIGHT CRC DO NOT DISTRIBUTE.

[27] Barry D, Hartigan JA (1993). A Bayesian analysis for change point problems. J Am Stat Assoc Taylor & Francis Group.

[28] Khaliq A, Ravindran R, Hussainy SF, Krishnan V V., Ambreen A, Yusuf NW, et al. Field evaluation of a blood based test for active tuberculosis in endemic settings. Cardona P-J, editor. PLoS One. Public Libr Sci; 2017;12:e0173359. 10.1371/journal.pone.0173359 PMC5381859 28380055

[29] Baccini M, Kosatsky T, Analitis A, Anderson HR, D’Ovidio M, Menne B (2011). Impact of heat on mortality in 15 European cities: attributable deaths under different weather scenarios. J Epidemiol Community Health.

[30] Rocklöv J, Forsberg B, Meister K (2009). Winter mortality modifies the heat-mortality association the following summer. Eur Respir J.

[31] Ha J, Kim H, Hajat S (2011). Effect of previous-winter mortality on the association between summer temperature and mortality in South Korea. Environ Health Perspect.

[32] Molbak K, Espenhain L, Nielsen J, Tersago K, Bossuyt N, Denissov G, et al. Excess mortality among the elderly in European countries, December 2014 to February 2015. Euro Surveill. 2015;20 10.2807/1560-7917.es2015.20.11.21065 25811643

[33] ECDC. In: Mereckiene J, editor. Seasonal influenza vaccination in Europe. Vaccination recommendations and coverage rates in the EU member states for eight influenza seasons. 2017th ed. Stockholm: European Centre for Disease Prevention and Control; 2017.

[34] Fouillet A, Rey G, Laurent F, Pavillon G, Bellec S, Guihenneuc-Jouyaux C (2006). Excess mortality related to the august 2003 heat wave in France. Int Arch Occup Environ Health.

[35] Green HK, Andrews N, Armstrong B, Bickler G, Pebody R (2016). Mortality during the 2013 heatwave in England--how did it compare to previous heatwaves? A retrospective observational study. Environ Res.

[36] Johnson H, Kovats RS, McGregor G, Stedman J, Gibbs M, Walton H (2005). The impact of the 2003 heat wave on daily mortality in England and Wales and the use of rapid weekly mortality estimates. Euro Surveill..

[37] Schulte E, Schmuck G, San-Miguel-Ayanz J, Barbosa P, Liberta G, European Communities (2002). Forest Fires in Europe - 2001 fire campaign.

[38] Analitis A, Georgiadis I, Katsouyanni K (2012). Forest fires are associated with elevated mortality in a dense urban setting. Occup Environ Med.

[39] Paldy A, Bobvos J (2010). Health Impacts of Heat Waves of 2007 In Hungary – background and experiences.

[40] Paldy A, Bobvos J. Health impacts of climate change in Hungary. Cent Eur J Occup Environ Med. 2014;20

[41] Grumm RH (2011). The central European and Russian heat event of July–august 2010. Bull Am Meteorol Soc American Meteorological Society.

[42] Kollanus V, Tiittanen P, Niemi JV, Lanki T (2016). Effects of long-range transported air pollution from vegetation fires on daily mortality and hospital admissions in the Helsinki metropolitan area, Finland. Environ Res.

[43] Irannezhad M, Chen D, Kløve B (2015). Interannual variations and trends in surface air temperature in Finland in relation to atmospheric circulation patterns, 1961-2011. Int J Climatol.

[44] Eurostat (2017). Population structure and ageing - Statistics Explained.

[45] Adeloye D, Chua S, Lee C, Basquill C, Papana A, Theodoratou E (2015). Global and regional estimates of COPD prevalence: Systematic review and meta-analysis. J Glob Health.

[46] Davis RE, Knappenberger PC, Novicoff WM, Michaels PJ (2003). Decadal changes in summer mortality in U.S. cities. Int J Biometeorol.

[47] Lee M, Nordio F, Zanobetti A, Kinney P, Vautard R, Schwartz J (2014). Acclimatization across space and time in the effects of temperature on mortality: a time-series analysis. Environ Health.

[48] Nordio F, Zanobetti A, Colicino E, Kloog I, Schwartz J (2015). Changing patterns of the temperature–mortality association by time and location in the US, and implications for climate change. Environ Int.

[49] Boeckmann M, Rohn I (2014). Is planned adaptation to heat reducing heat-related mortality and illness?. A systematic review BMC Public Health.

[50] Sheridan SC, Allen MJ (2018). Temporal trends in human vulnerability to excessive heat. Environ Res Lett..

[51] Hondula DM, Balling RC, Vanos JK, Georgescu M (2015). Rising Temperatures, Human Health, and the Role of Adaptation. Curr Clim Chang Reports.

[52] Bittner M-I, Matthies EF, Dalbokova D, Menne B (2014). Are European countries prepared for the next big heat-wave?. Eur J Pub Health.

[53] Lowe D, Ebi KL, Forsberg B (2011). Heatwave early warning systems and adaptation advice to reduce human health consequences of heatwaves. Int J Environ Res Public Health.

[54] Matthies F, Bickler G, HS CMN, WHO Regional Office for Europe (2008). Heat-Health Action Plans - Guidance.

[55] Michelozzi P, de’Donato FK, Bargagli AM, D’Ippoliti D, De Sario M, Marino C (2010). Surveillance of summer mortality and preparedness to reduce the health impact of heat waves in Italy. Int J Environ Res Public Health.

[56] Bhatnagar P, Wickramasinghe K, Wilkins E, Townsend N (2016). Trends in the epidemiology of cardiovascular disease in the UK. Heart BMJ Publishing Group Ltd and British Cardiovascular Society.

[57] Christidis N, Donaldson GC, Stott PA (2010). Causes for the recent changes in cold- and heat-related mortality in England and Wales. Clim Change Springer Netherlands.

