# Supplementary figures and images for: The inter-annual variability of heat-related mortality in nine European cities (1990–2010)
Source: Environ Health. 2018 Aug 8;17:66. doi: 10.1186/s12940-018-0411-0 (PMC6083580; doi:10.1186/s12940-018-0411-0)

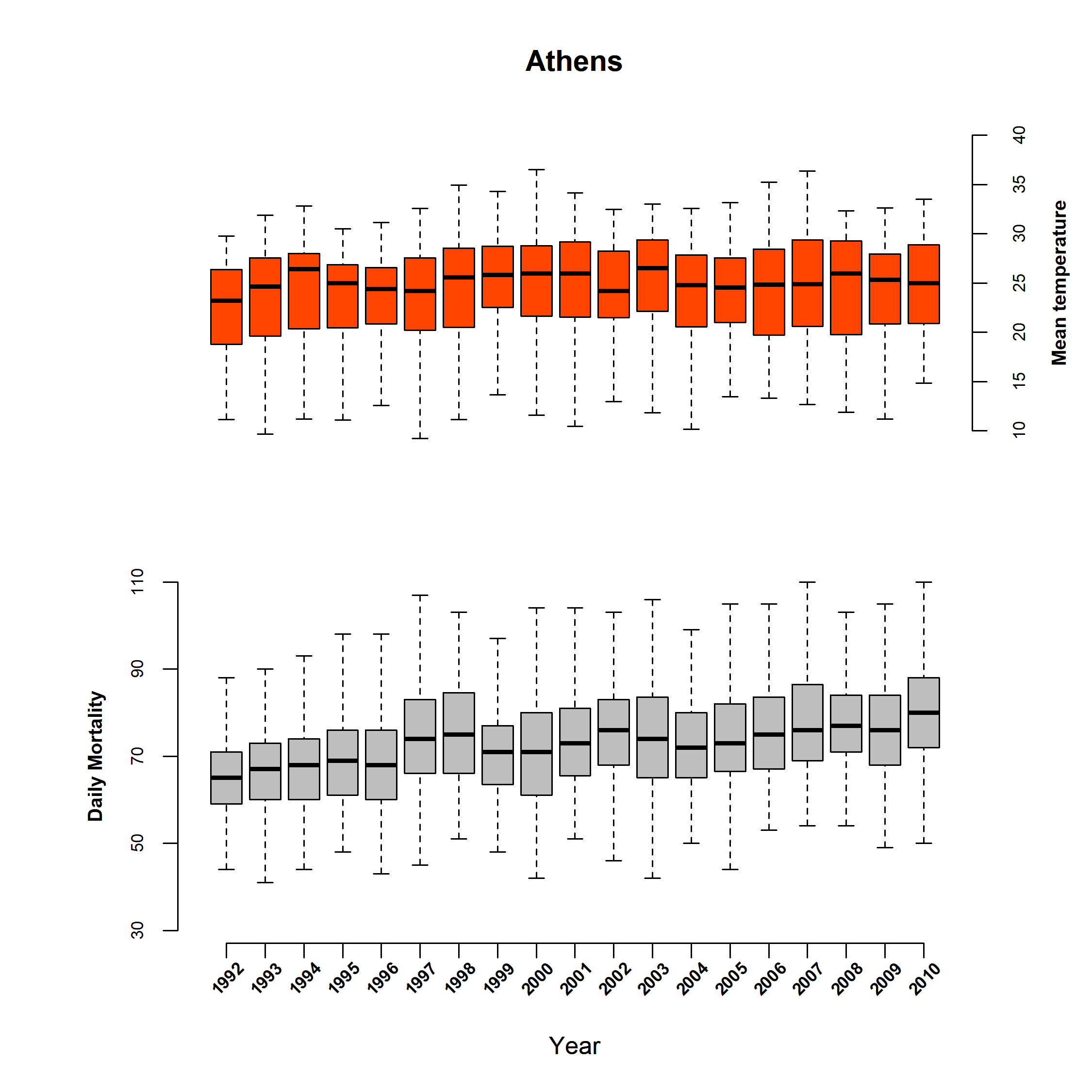

Supplement: Supplementary file 2 — Figures S2-S10. Temperature and mortality distribution by year in the nine European cities (period 1990–2010). Boxplots of temperature and mortality for each city and for each year. (ZIP 934 kb) [file 12940_2018_411_MOESM2_ESM.zip › 12940_2018_411_MOESM10_ESM.tif]

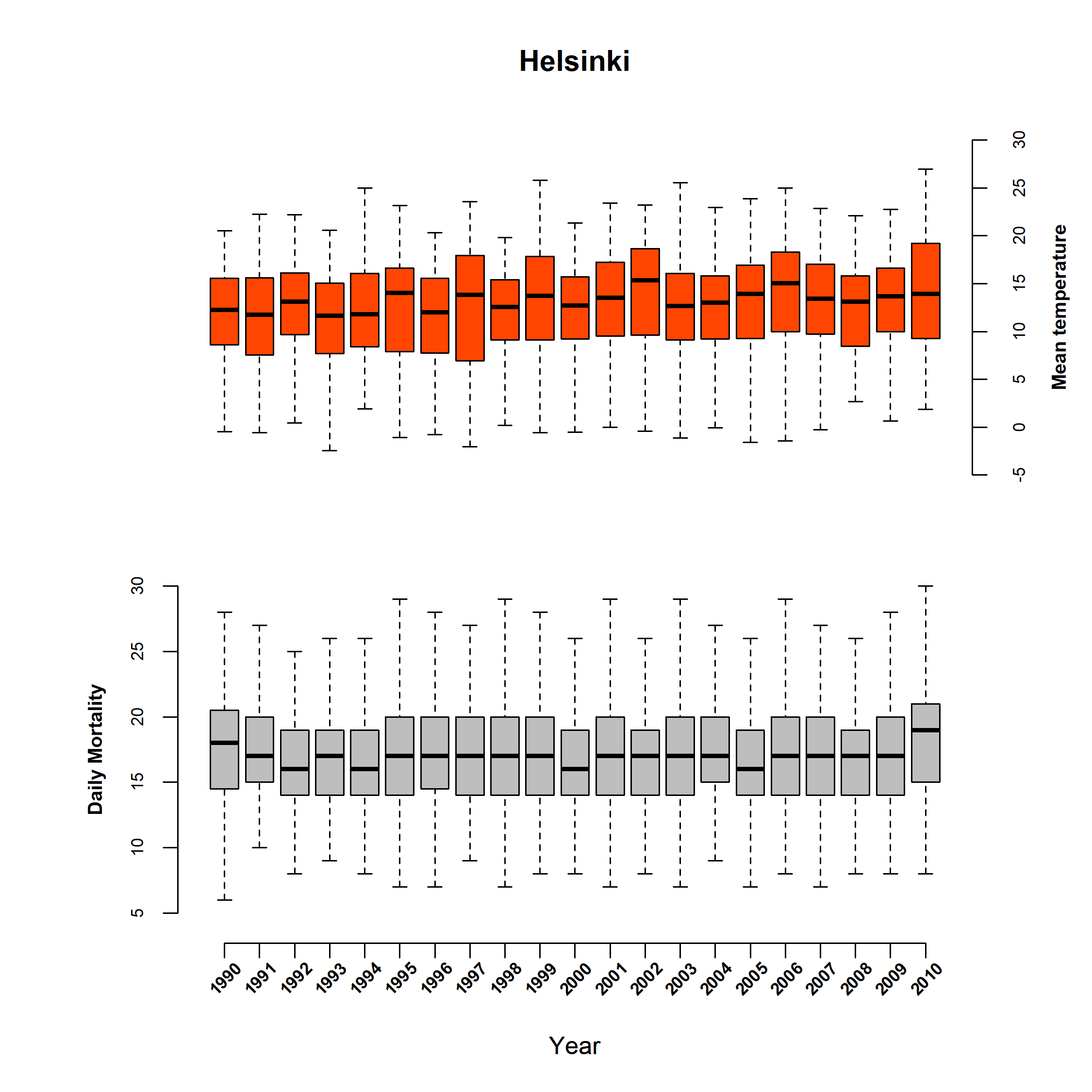

Supplement: Supplementary file 2 — Figures S2-S10. Temperature and mortality distribution by year in the nine European cities (period 1990–2010). Boxplots of temperature and mortality for each city and for each year. (ZIP 934 kb) [file 12940_2018_411_MOESM2_ESM.zip › 12940_2018_411_MOESM2_ESM.tif]

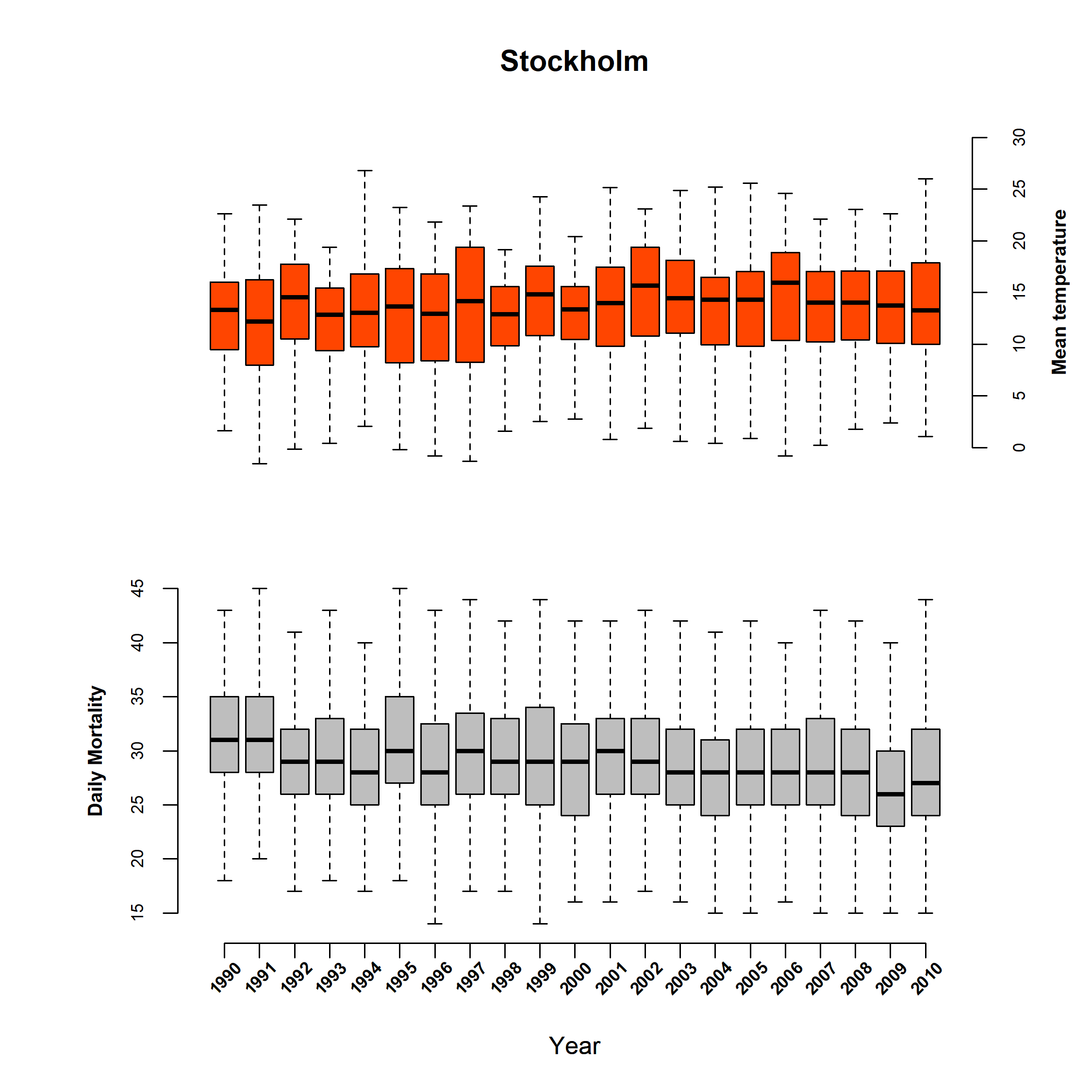

Supplement: Supplementary file 2 — Figures S2-S10. Temperature and mortality distribution by year in the nine European cities (period 1990–2010). Boxplots of temperature and mortality for each city and for each year. (ZIP 934 kb) [file 12940_2018_411_MOESM2_ESM.zip › 12940_2018_411_MOESM3_ESM.tif]

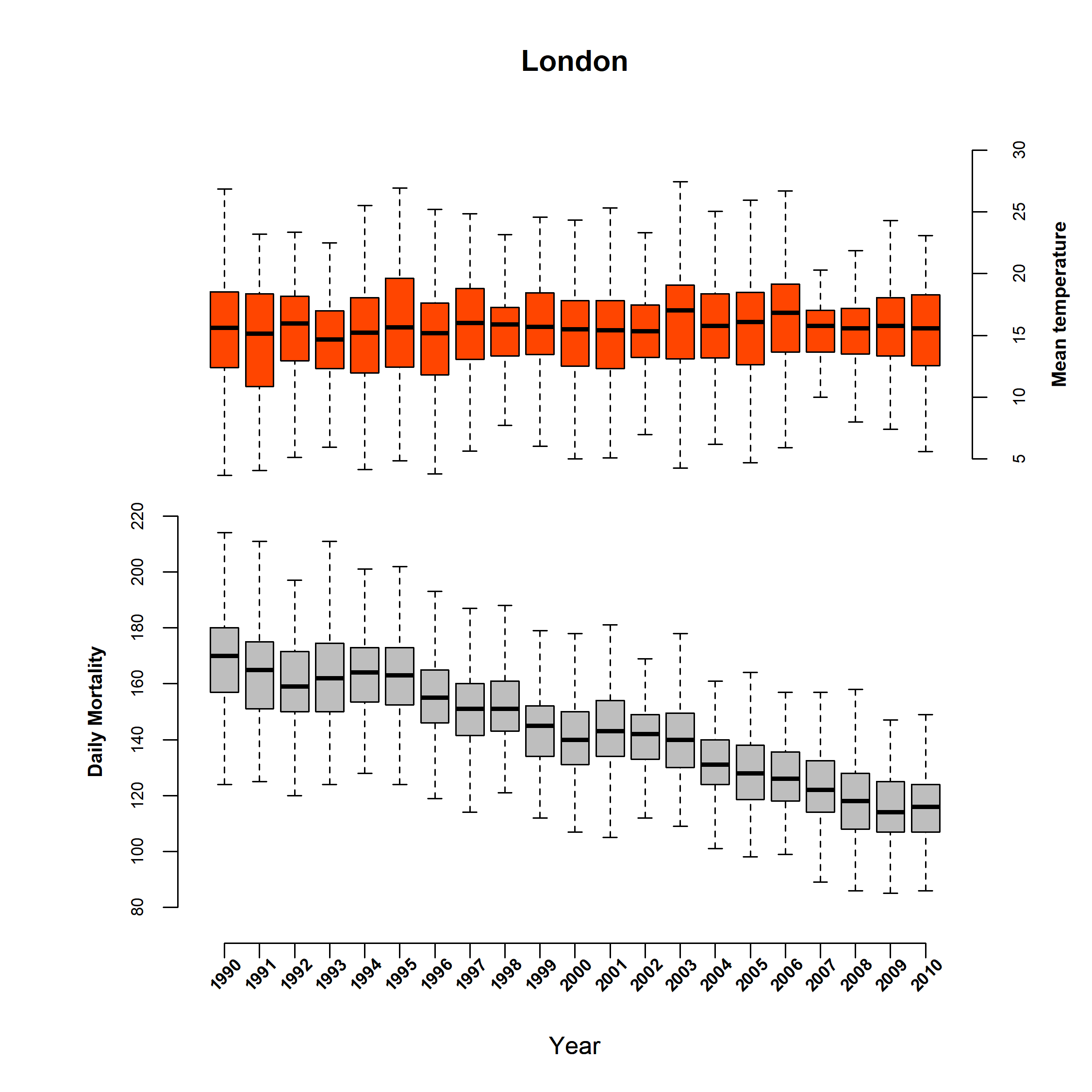

Supplement: Supplementary file 2 — Figures S2-S10. Temperature and mortality distribution by year in the nine European cities (period 1990–2010). Boxplots of temperature and mortality for each city and for each year. (ZIP 934 kb) [file 12940_2018_411_MOESM2_ESM.zip › 12940_2018_411_MOESM4_ESM.tif]

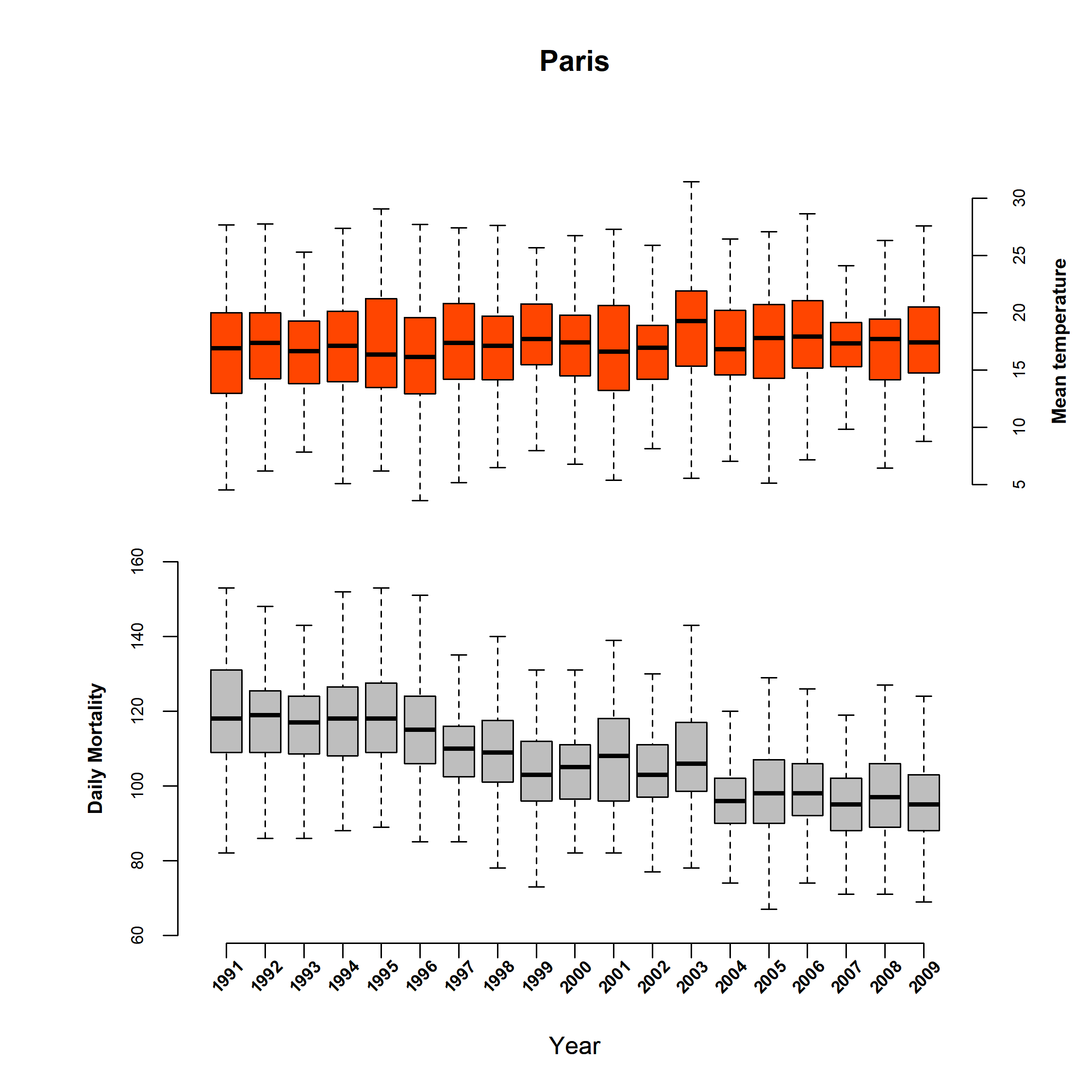

Supplement: Supplementary file 2 — Figures S2-S10. Temperature and mortality distribution by year in the nine European cities (period 1990–2010). Boxplots of temperature and mortality for each city and for each year. (ZIP 934 kb) [file 12940_2018_411_MOESM2_ESM.zip › 12940_2018_411_MOESM5_ESM.tif]

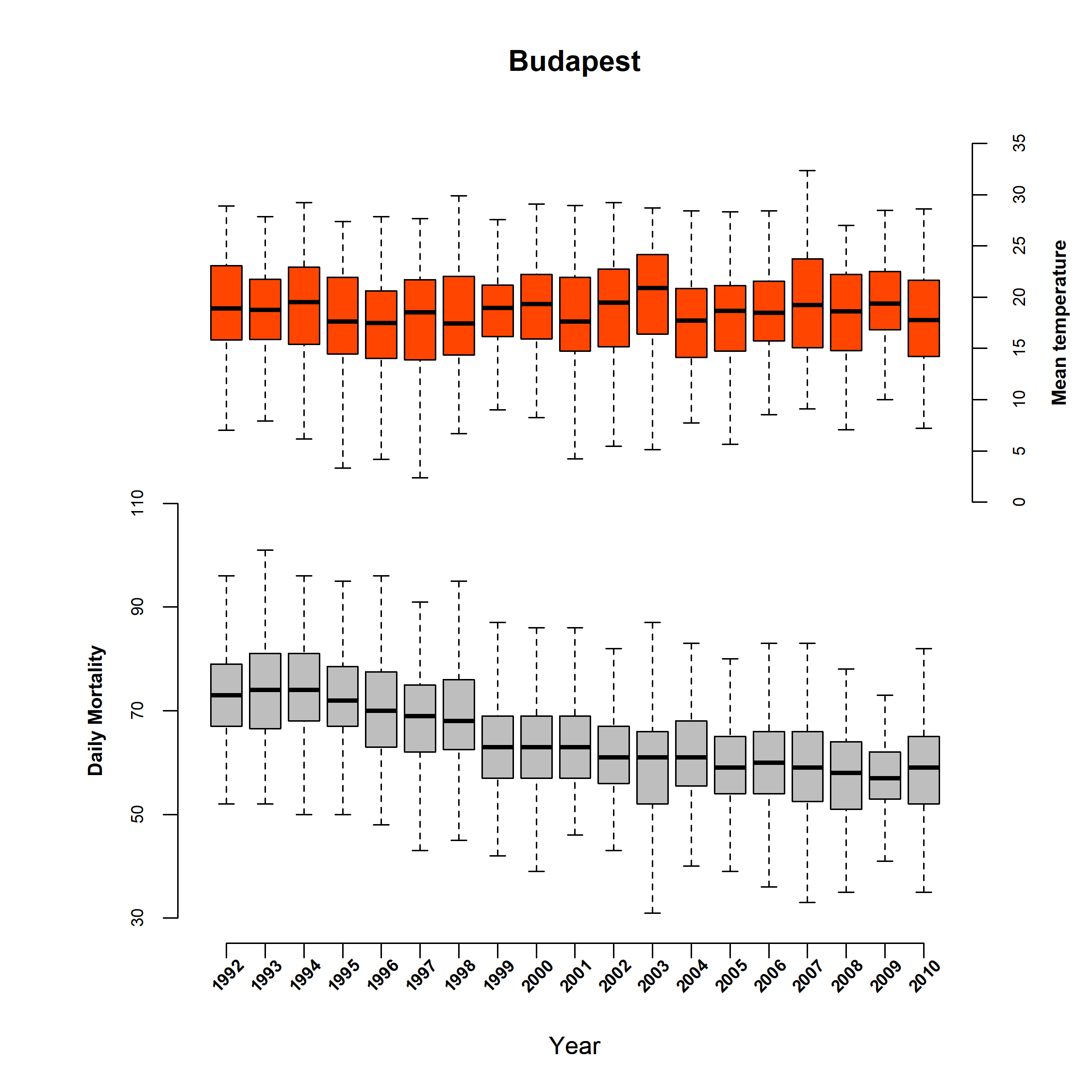

Supplement: Supplementary file 2 — Figures S2-S10. Temperature and mortality distribution by year in the nine European cities (period 1990–2010). Boxplots of temperature and mortality for each city and for each year. (ZIP 934 kb) [file 12940_2018_411_MOESM2_ESM.zip › 12940_2018_411_MOESM6_ESM.tif]

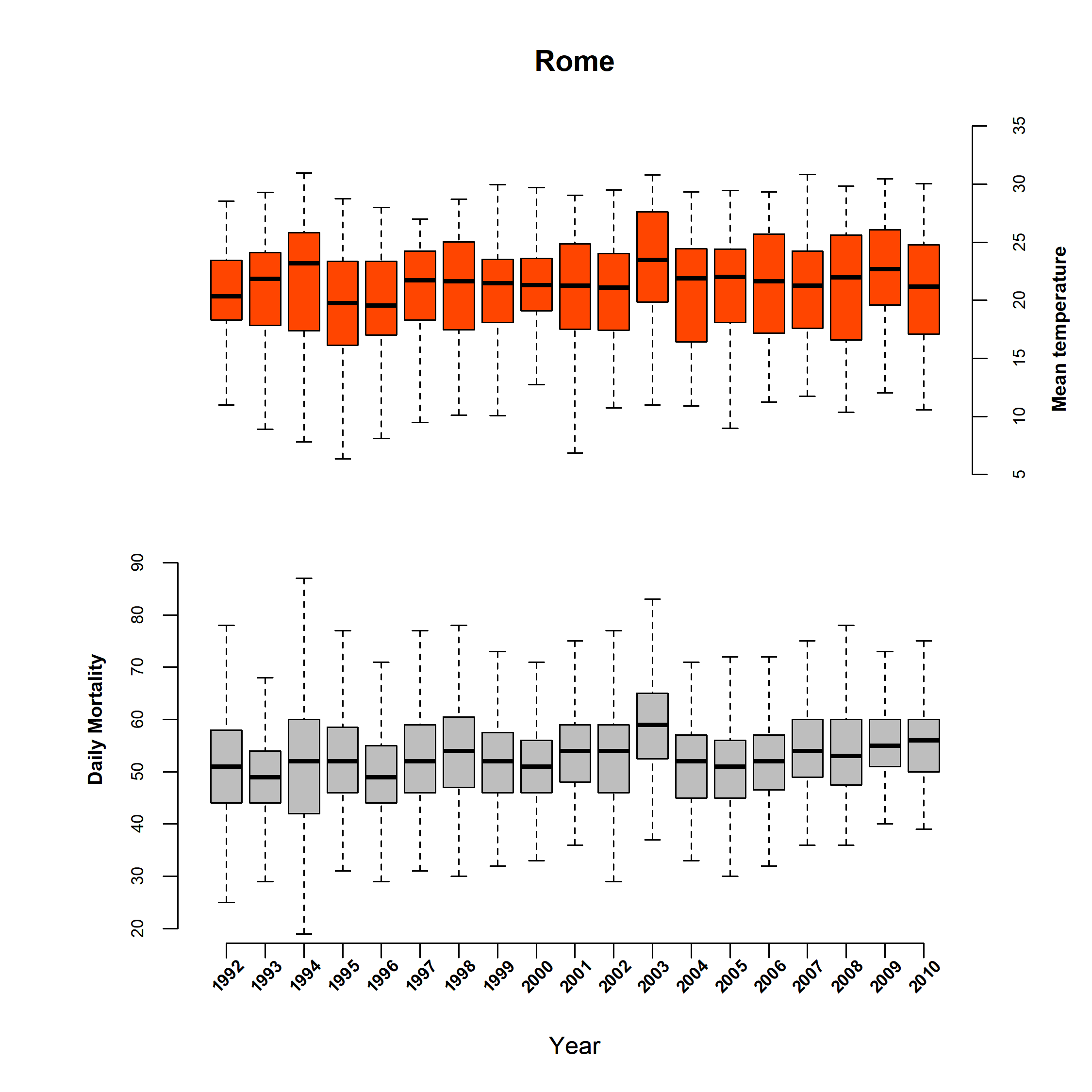

Supplement: Supplementary file 2 — Figures S2-S10. Temperature and mortality distribution by year in the nine European cities (period 1990–2010). Boxplots of temperature and mortality for each city and for each year. (ZIP 934 kb) [file 12940_2018_411_MOESM2_ESM.zip › 12940_2018_411_MOESM7_ESM.tif]

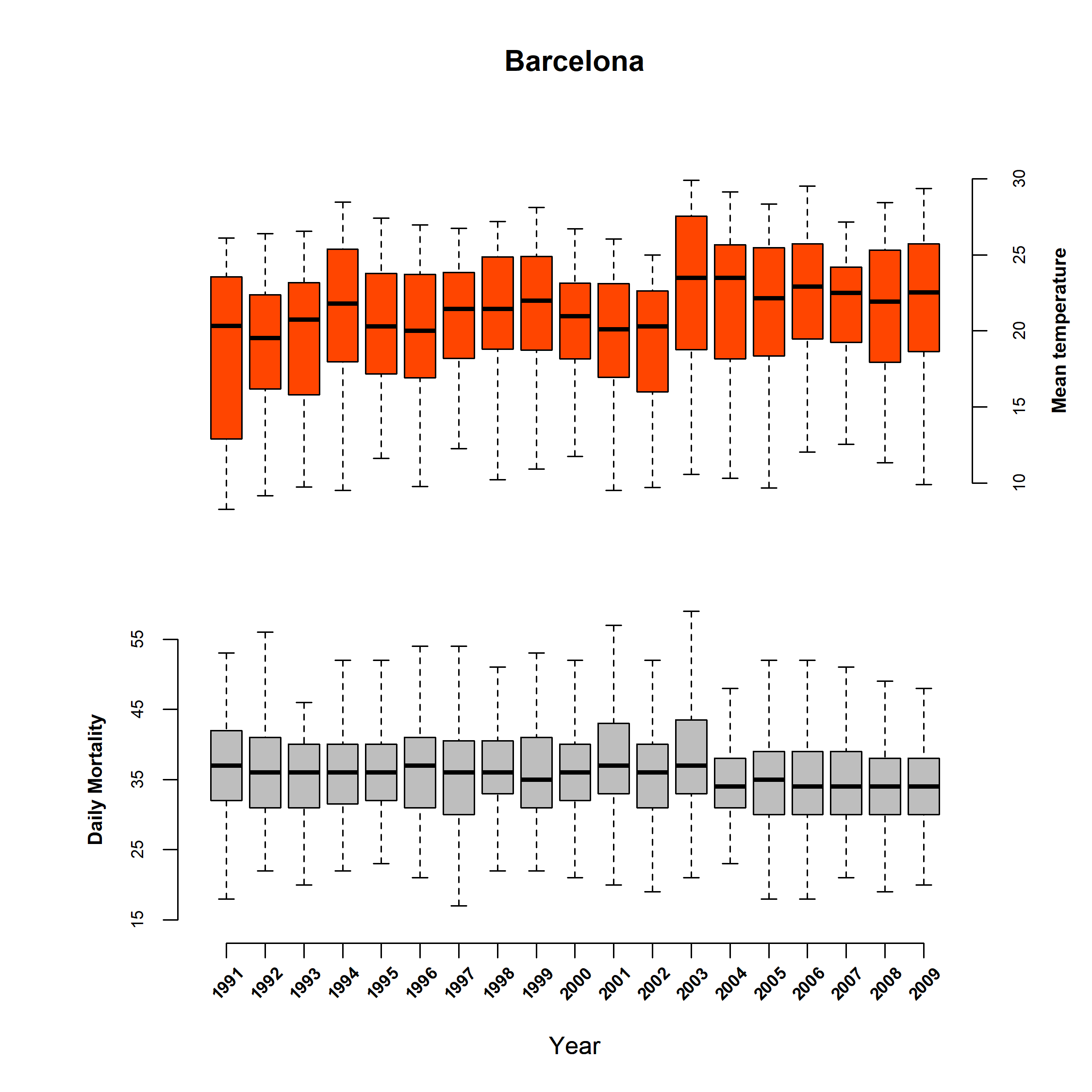

Supplement: Supplementary file 2 — Figures S2-S10. Temperature and mortality distribution by year in the nine European cities (period 1990–2010). Boxplots of temperature and mortality for each city and for each year. (ZIP 934 kb) [file 12940_2018_411_MOESM2_ESM.zip › 12940_2018_411_MOESM8_ESM.tif]

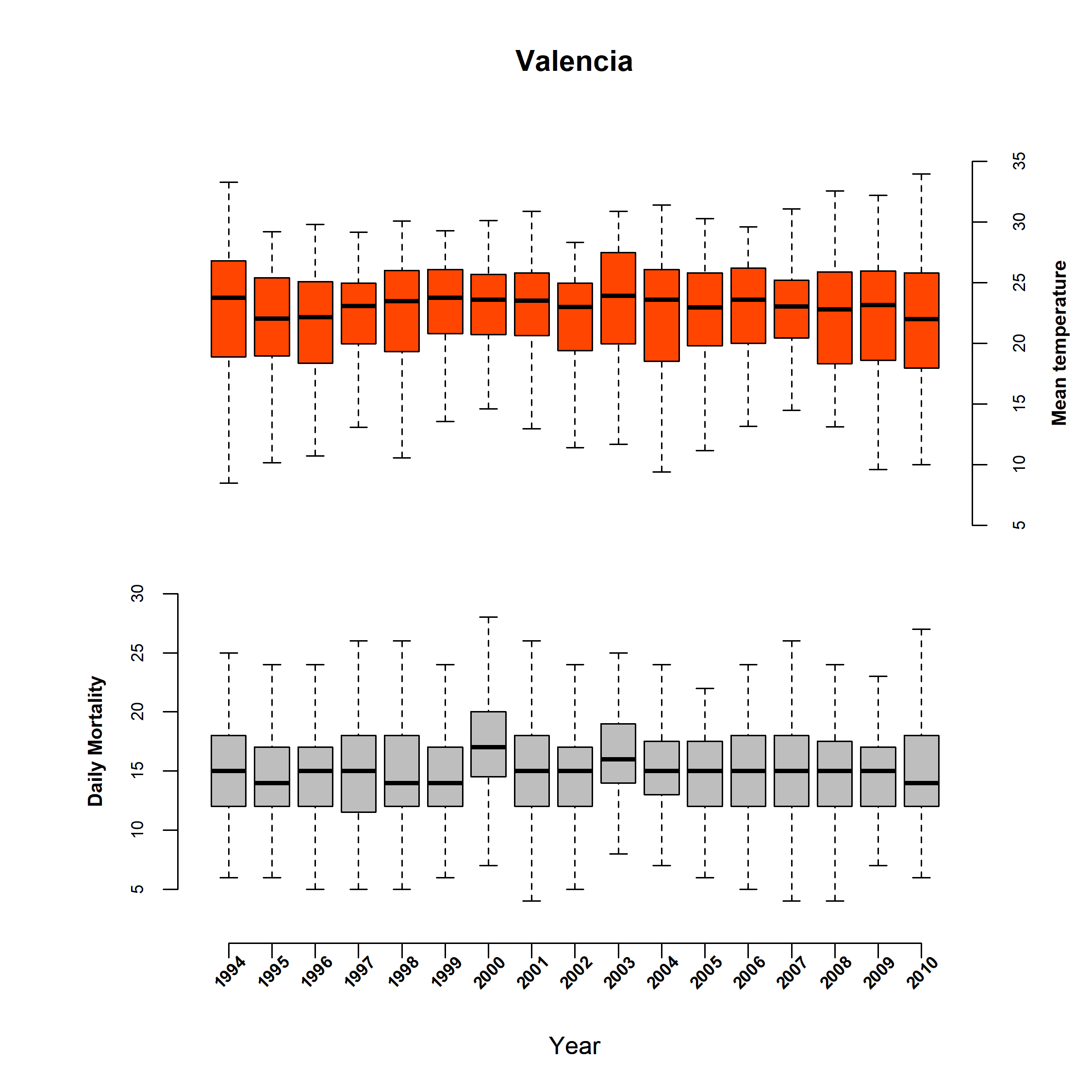

Supplement: Supplementary file 2 — Figures S2-S10. Temperature and mortality distribution by year in the nine European cities (period 1990–2010). Boxplots of temperature and mortality for each city and for each year. (ZIP 934 kb) [file 12940_2018_411_MOESM2_ESM.zip › 12940_2018_411_MOESM9_ESM.tif]
